# Mismatch Repair Gene MLH3 Pro844Leu and Thr942Ile Polymorphisms and the Susceptibility to Cervical Carcinoma and HPV Infection: A Case-Control Study in a Chinese Population

**DOI:** 10.1371/journal.pone.0096224

**Published:** 2014-04-23

**Authors:** Feng Ye, Qi Cheng, Jiajie Shen, Caiyun Zhou, Huaizeng Chen

**Affiliations:** 1 Women’s Reproductive Health Key Laboratory of Zhejiang Province, Women’s Hospital, School of Medicine, Zhejiang University, Hangzhou, Zhejiang, China; 2 Department of Pathology, Women’s Hospital, School of Medicine, Zhejiang University, Hangzhou, Zhejiang, China; University of North Carolina School of Medicine, United States of America

## Abstract

To investigate the association between MLH3 Pro844Leu, Thr942Ile polymorphisms and potential linkage with the risk of cervical carcinoma and potential effect on protein function, we carried out a case-control study with 400 cervical squamous cell carcinoma, 400 CIN3 and 1200 normal controls in a Chinese population. The results showed that there was an increased risk of cervical carcinoma and CIN3 associated with the genotype 844CT [OR 2.17 (1.61–2.94); *P*<0.001; OR 1.49 (1.08–2.07), *P* 0.017, respectively] and a decreased risk with the 942CT genotype [OR 0.56 (0.38–0.82); *P*<0.001; OR 0.37 (0.24–0.58), *P*<0.001, respectively]. Most 844CT genotypes were linkage CT(844)-CC(942), which increased the risk of cervical carcinoma and CIN3 [77/83, OR 2.04 (1.48–2.80), *P*<0.001; 55/61, OR 1.46 (1.03–2.06), *P* 0.035, respectively]. Most 942CT were linkage CC(844)-CT(942), which decreased the risk of cervical carcinoma [29/35, OR 0.60 (0.40–0.91); *P* 0.017; 18/24, OR 0.33 (0.20–0.55), *P*<0.001, respectively]. In some grouping, the 844CT and 942CT were further enriched; especially HR-HPV-positive subjects both in the CIN3 and the cervical carcinoma, the 844CT had greater enrichment. These results included that CT(844)-CC(942) was associated with a high risk of cervical carcinoma and CIN3, and the CC(844)-CT(942) decreased the risk. The 844CT had a higher level of enrichment in HR-HPV positive individuals, which is probably related to HR-HPV susceptibility. There was no significant difference of the MLH3 mRNA expression and these two amino acid substitutions did not impact on the protein function.

## Introduction

Cervical carcinoma is the second most common type of cancer among women worldwide [1]. HPV is regarded as a prime etiologic factor in cervical carcinoma and its precursor lesion cervical intraepithelial neoplasia (CIN) [2]. Although ∼30% of sexually active women are infected with high risk human papillomavirus (HR-HPV), few of women infected with HR-HPV (∼1%) will develop CIN and cervical carcinoma [3]. This indicates HPV infection is necessary but insufficient for the cervical carcinogenesis. Consequently, abnormalities in cell repair machinery may be a co-factor in addition to HPV infection for malignancy development. Tumorigenesis is the result of intrinsic and environmental factors; the main environmental factor in cervical cancer is HR-HPV infection and the presence of abnormal genetic background is the main internal factor of concern. This is reflected mainly in genome instability, which can be induced by the lack of genetic error repair and correction.

The DNA mismatch repair (MMR) system has a crucial role in genomic stability by correcting errors acquired during DNA replication and recombination [4]. Malfunction of the MMR results in replication of an error-positive genotype and microsatellite instability [5]. Cells with defective MMR function show a mutator phenotype characterized by increased DNA mutation rate leading to increased susceptibility to cancer [6]. MMR genes have attracted of attention because of their association with hereditary nonpolyposis colorectal cancer (HNPCC) [7]. The MMR system consists of DNA-binding components (MSH2, MSH3 and MSH6) and DNA repair components (MLH1 and PMS1) [8]. These MMR genes have been found to be mutated in HNPCC and in other cancers [7], [8]. The MMR gene, MLH3, is located on 14q24.3 with a coding length of 4.3 kb [9]. To date, 22 variants have been reported, 12 of which are missense changes, including Pro844Leu and Thr942Ile [10].

MLH3 might have a role in colorectal cancer tumorigenesis [11]. MLH3 polymorphisms are reported to be related to the risk of lung and breast cancer [12], [13]. These suggest the MLH3 mutation might be involved in the development of cancer. Although a low incidence of microsatellite instability was reported in patients with cervical carcinomas [14], the MMR genes Msh2 and Mlh1 are associated with a higher risk for cervical carcinomas [15]. Thus, the MMR system might be important in the development of cervical carcinomas. Our earlier study found some DNA repair genes single nucleotide polymorphism (SNP), for example PARP-1, XRCC3 and XPD, were associated with cervical carcinogenesis and HR-HPV infection [16], [17]. This prompted us to undertake further study of the relationship between MMR genes, SNP and the susceptibility to cervical carcinoma.

In this study, we investigated the distribution of the common Pro844Leu and Thr942Ile genotypes in Chinese women, and assessed the association of these polymorphisms or linkage disequilibrium with the risk of cervical squamous cell carcinoma (CSCC), CIN III and their interaction with HR-HPV infection, sexual and reproductive risk factors, and analyzed the MLH3 gene mRNA expression level and the effect on protein function.

## Materials and Methods

### Participants

400 CSCC patients, 400 CIN III patients and 1200 normal controls were recruited from Zhejiang Province, China. The diagnosis was confirmed by two pathologists. Normal controls were collected from healthy women volunteers during gynecologic examinations between June 2004 and December 2008. The selection criteria for healthy controls included no positive cytological finding, no gynecological tumor, no endometriosis, no other cancer history, no immune disease. Of these, 201 patients with CSCC, 357 patients with CIN III and 637 controls agreed to provide cervical brush-off samples for HR-HPV test.

This study was approved by the Medical Ethical Committee of Women’s Hospital, School of Medicine, Zhejiang University (No.2004002). All patients signed informed consent to allow molecular research on specimen obtained.

### DNA Extraction and Genotyping

Genomic DNA was extracted from peripheral blood using a DNA extraction kit (Sangon Bioengineering Co., Shanghai, China). All DNA samples were dissolved in water and stored at –20°C.

MLH3 Pro844Leu (CCT2645CTT) or Thr942Ile (ACA2939ATA) genotypes were determined by bidirectional sequencing. The forward and reverse primers for MLH3 were 5′-CAGAAGTAGAGGAAAGTAATGG-3′ and 5′-CTGTGGCATCTTCTACCG-3′, respectively, which produce a 732 bp fragment. PCR was done in a 25 µl reaction mixture. The reaction conditions were: 94°C 5 min, 35cycles of 94°C 30 s, 55°C 30 s, 72°C for 45 s and 72°C for 5 min.

The PCR product was purified and amplified with a BigDye terminator 3.1 kit (Applied Biosystems, USA). Amplified dye-terminated products were sequenced using forward and reverse primers with the ABI PRISM 3100 Genetic Analyzer.

### HR-HPV Detection

HR-HPV infection was assayed using the HC II kit (Digene Diagnostics Inc., Gaitherburg, MD, USA). Cervical sampling for HPV DNA was done with the Digene Cervical Sampler by measuring the relative light unit (RLU)/positive control ratio:RLU of specimen/mean RLU of two positive controls.

### Total RNA Extraction and qRT-PCR

Histological examination of 282 freshly frozen tumor tissue samples confirmed cervical squamous cell carcinoma. Total RNA was extracted by using TRIzol reagent (Invitrogen, USA) and subjected to reverse transcription after digestion with RNase-free DNase I. The following primers were used for qRT-PCR; 95°C 30 s, 40 cycles at 95°C 5 s followed with 60°C 35 s.

MLH3 primers:

forward: 5′-TGCCCGTTATCCAGAGGTTG-3′.

reverse: 5′-TATACGCTCATGGGCAGCG-3′.

GAPDH primers:

forward: 5′-GAGAAGGCTGGGGCTCATTT-3′.

reverse: 5′-AGTGATGGCATGGACTGTGG-3′.

The MLH3 and GAPDH PCR products were 244 bp and 231 bp, respectively. qRT-PCR was done with the SYBR Premix Ex Taq kit(Takara Biotechnology, Japan) with an Applied Biosystems 7900 HT system. All samples were run in triplicate. The relative mRNA expression levels were calculated according to the 2–Δ*C*_t_ method, in which Δ*C*_t_ is the difference in value between each *C*_t_ value of the target gene minus that of GAPDH.

### In silico Analysis of Amino Acid Substitutions

Predicting the effects of nonsynonymous SNPs on protein function was done with the SIFT algorithm (http://sift.bii.a-star.edu.sg). Amino acid substitutions are reported as TOLERATED or DAMAGING. SIFT scores were designated as tolerant (0.201–1.00), borderline (0.101–0.20), potentially intolerant (0.051–0.10) or intolerant (0.00–0.05).

### Statistical Analysis

Differences in lifestyle habits and genotype frequencies were evaluated with Pearson’s χ^2^ test. For the association between the genotypes and risk of cervical carcinoma, the odds ratio (OR), 95% confidence intervals(CIs) and *P*-values were obtained by binary logistic regression analysis. The control was set as the reference group for analysis. For mRNA expression analysis, we used the 1-sample *KS* nonparametric test to detect the normal distribution of 2^–Δ*C*t^ values and Levene’s test for equality of variances to verify the homogeneity of variance and then we chose the independent sample *t*-test to define the differences.

All reported values are two-tailed. The level of statistically significant difference was set at *P*≤0.05. All statistical analysis was done with SPSS for Windows software 10.0 version.

## Results

### Genomic DNA, PCR and Polymorphism Sequencing Quality Control (Figures 1A–F)

The genomic DNA showed a single band corresponding to a length of 30∼50 kb, which indicated it was not degraded. There was sufficient PCR product for sequencing and each showed a single band. The sequencing results gave sharp peaks with no significant overlap, which showed the nucleotide sequence was very reliable.

### Clinical Characteristics of Patients and Controls (Table 1)

In the control, CIN III and carcinoma groups, 602/598, 258/142 and 160/240 individuals were >40 years old/<40 years old, respectively. The carcinoma group had significantly more individuals >40 years old (*P*<0.001) and the CIN III group had significantly more individuals <40 years old (*P*<0.001) compared to the control. There was no significant difference beside the *increase of the proportion of individuals with* number of parities >3 in *both the* carcinoma *and CIN* III *groups*. The HR-HPV infection rate was 88.6% in the carcinoma and 86.8% in the CIN III but only 31.4% in the control, which indicated the infection rate in both patient groups was significantly higher compared to the control (*P*<0.001). These indicated that smoking and HR-HPV infection in CIN III and cervical carcinoma patients were more than controls.

**Table 1:** Frequency distribution of select characteristics by case control status.

Variable		Control	CIN III	χ*^2a^*	*P*	carcinoma	χ*^2a^*	P
		n = 1200	n = 400			n = 400		
		n(%)	n(%)			n(%)		
Age	≤40	602(50.2)	258(64.5)	**24.793**	**<0.001**	160(40.0)	**12.431**	**<0.001**
	>40	598(49.8)	142(35.5)			240(60.0)		
Number of sexual partners	≤1	963(80.3)	316(79.0)	0.292	0.589	309(77.3)	1.657	0.198
	>1	237(19.8)	84(21.0)			91(22.8)		
Age at the first intercourse	≤20years	359(29.9)	130(32.5)	0.943	0.331	125(31.3)	0.253	0.615
	>20years	841(70.1)	270(67.5)			275(68.8)		
Number of parities^b^	≤3	548(45.7)	158(39.5)	**4.627**	**0.031**	131(32.8)	**20.49**	**<0.001**
	>3	652(54.3)	242(60.5)			269(67.3)		
Age at the first birth	≤22years	235(19.6)	91(22.8)	1.854	0.173	89(22.3)	1.321	0.25
	>22years	965(80.4)	309(77.3)			311(77.8)		
Smoking status	smoker	4(0.3)	2(0.5)	0.223	0.637	2(0.5)	0.223	0.637
	nonsmoker	1196(99.7)	398(99.5)			398(99.5)		
HR-HPV infection	Positive	191(31.4)	310(86.8)	**277.107**	**<0.001**	178(88.6)	**199.315**	**<0.001**
	Negative	418(68.6)	47(13.2)			23(11.4)		
	total	609	357			201		

Bold values show statistical data with significant difference.

a Two-tailed χ^2^ test.

b Parities including full-term pregnancy and abortion at or after 28 weeks.

### Case–control Single Analysis: Association between MLH3 Pro844Leu, Thr942Ile and Risk of CSCC (Table 2)

The frequency of Pro844Pro(844CC) and Pro844Leu(844CT) was 89.3% and 10.8% in the control, 84.8% and 15.3% in the CIN III and 79.3% and 20.8% in CSCC, respectively. These results revealed that women with the heterozygous Pro844Leu (844CT) genotype had a significantly increased risk of CIN III [OR 1.49 (1.08–2.07); *P* 0.017] and CSCC [OR 2.17 (1.61–2.94); *P*<0.001]. The frequency of the Thr942Ile (942CT) genotype was significantly different between patients and controls. Women heterozygous for the Thr942Ile (942CT) genotype had a decreased risk of CIN III [OR 0.37(0.24–0.58); *P*<0.001] and CSCC [OR 0.56 (0.38–0.82); *P*<0.001]. In the HR-HPV positive group, the heterozygous Pro844Leu (844CT) genotype increased the risk of CSCC significantly [OR 3.06(1.67–5.62); *P*<0.001]. The heterozygous Thr942Ile (942CT) genotype might decrease the risk of CIN III[OR 0.53(0.28–1.01; *P* 0.054], whereas the Thr942Ile(942CT) genotype did not decrease the risk of CSCC[OR 0.75 (0.37–1.50); *P* 0.407].

**Table 2:** Association between MLH3 polymorphisms and the risk of cervical squamous cell carcinoma and CIN III in all and HPV-positive subjects.

	All patients and controls							HPV-positive patients and controls						
MLH3 genotypes	Control, n = 1200	CIN III, n = 400	adjusted OR* (95% CI)	P	carcinoma, n = 400	adjusted OR* (95% CI)	P	Control, n = 191	CIN III, n = 310	adjusted OR* (95% CI)	P	carcinoma, n = 178	adjusted OR* (95% CI)	P
	n(%)	n(%)			n(%)			n(%)	n(%)			n(%)		
Pro844Leu														
CC	1071(89.3)	339(84.8)	1.00		317(79.3)	1.00		174(91.1)	265(85.5)	1.00		37(77.0)	1.00	
CT	129(10.8)	61(15.3)	**1.49(1.08–2.07)**	**0.017**	83(20.8)	**2.17(1.61–2.94)**	**<0.001**	17(8.9)	45(14.5)	1.74(0.96**–**3.13)	0.067	41(23.0)	**3.06(1.67–5.62)**	**<0.001**
Thr942Ile														
CC	1025(85.4)	376(94.0)	1.00		365(91.3)	1.00		170(89.0)	291(93.9)	1.00		163(91.6)	1.00	
CT	175(14.6)	24(6.0)	**0.37(0.24–0.58)**	**<0.001**	35(8.8)	**0.56(0.38–0.82)**	**<0.001**	21(11.0)	19(6.1)	**0.53(0.28–1.01)**	0.054	15(8.4)	0.75(0.37**–**1.50)	0.407

Bold values show statistical data with significant difference.

*All P-values are adjusted for age, number of sexual partners, age at first intercourse, parities (including full-term pregnancy and abortion at or after 28 weeks) and age at first full-term pregnancy.

### Association between MLH3 Pro844Leu, Thr942Ile and the Sexual, Reproductive History in CSCC and CIN III (Table 3)

The participants were divided into two groups according to (1) number of sexual partners, (2) age of first sexual intercourse, (3) number of parities and (4) age at first parity and then we analyzed the association with the Pro844Leu (844CT) genotype. We did not find a particularly high level of enrichment between groups, because, *P* was <0.05 or >0.05 in the statistics for any group. Apart from the number of sexual partners in the CIN III, there was a particularly high level of enrichment because *P* of more than 1 group was >0.05 (*P* 0.454) whereas those of the other groups were <0.05(*P* 0.022).

**Table 3:** Association between the MLH3 Pro844Leu, Thr942Ile genotype and the risk for CIN III and cervical squamous cell carcinoma stratified by various environmental factors.

Loci	High risk exposure		Controls		CIN III		Adjusted OR*(95% CI)	P	Carcinoma		Adjusted OR* (95% CI)	P
			CC(%)	CT(%)	CC(%)	CT(%)			CC(%)	CT(%)		
Pro844Leu	Number of sexual partners	≤1	858(89.1)	105(10.9)	266(84.2)	50(15.8)	**1.53(1.06–2.20)**	**0.022**	245(79.3)	64(20.7)	**2.14(1.52–3.00)**	**<0.001**
		>1	213(89.9)	24(10.1)	73(86.9)	11(13.1)	1.34(0.62–2.86)	0.454	72(79.1)	19(20.9)	**2.34(1.21–4.53)**	**0.011**
	Age at the first intercourse	≤20	321(89.4)	38(10.6)	110(84.6)	20(15.4)	1.54(0.86–2.75)	0.149	97(77.6)	28(22.4)	**2.44(1.42–4.18)**	**0.001**
		>20	750(89.2)	91(10.8)	229(84.8)	41(15.2)	1.48(0.99–2.20)	0.055	220(80.0)	55(20.0)	**2.06(1.43–2.97)**	**<0.001**
	Number of parities	≤3	490(89.4)	58(10.6)	133(84.2)	25(15.8)	1.59(0.96–2.64)	0.074	103(78.6)	28(21.4)	**2.30(1.40–3.78)**	**0.001**
		>3	581(89.1)	71(10.9)	206(85.1)	36(14.9)	1.43(0.93–2.20)	0.104	214(79.6)	55(20.4)	**2.10(1.43–3.09)**	**<0.001**
	Age at the first parity	≤22	214(91.1)	21(8.9)	77(84.6)	14(15.4)	1.85(0.90–3.82)	0.095	72(80.9)	17(19.1)	**2.41(1.20–4.81)**	**0.013**
		>22	857(88.8)	108(11.2)	262(84.8)	47(15.2)	1.42(0.98–2.06)	0.061	245(78.8)	66(21.2)	**2.14(1.53–3.00)**	**<0.001**
	HR-HPV infection status	+	174(91.1)	17(8.9)	265(85.5)	45(14.5)	1.74(0.96–3.13)	0.067	137(77.0)	41(23.0)	**3.06(1.67–5.62)**	**<0.001**
		-	381(91.1)	37(8.9)	41(87.2)	6(12.8)	1.51(0.60–3.78)	0.383	20(87.0)	3(13.0)	1.55(0.44–5.44)	0.500
Thr942Ile	Number of sexual partners	≤1	822(85.4)	141(14.6)	298(94.3)	18(5.7)	**0.35(0.21–0.59)**	**<0.001**	285(92.2)	24(7.8)	**0.49(0.31–0.77)**	**0.002**
		>1	203(85.7)	34(14.3)	78(92.9)	6(7.1)	0.46(0.19–1.14)	0.093	80(87.9)	11(12.1)	0.82(0.40–1.70)	0.600
	Age at the first intercourse	≤20	210(86.4)	49(13.6)	126(96.9)	4(3.1)	**0.14(0.05–0.39)**	**<0.001**	117(93.6)	8(6.4)	**0.29(0.13–0.64)**	**0.002**
		>20	715(85.0)	126(15.0)	250(92.6)	20(7.4)	**0.45(0.28–0.74)**	**0.002**	248(90.2)	27(9.8)	**0.62(0.40–0.96)**	**0.032**
	Number of parities	≤3	477(87.0)	71(13.0)	154(97.5)	4(2.5)	**0.18(0.06–0.49)**	**0.001**	119(90.8)	12(9.2)	0.68(0.36–1.29)	0.236
		>3	548(84.0)	104(16.0)	222(91.7)	20(8.3)	**0.48(0.29–0.79)**	**0.004**	246(91.4)	23(8.6)	**0.49(0.31–0.79)**	**0.004**
	Age at the first parity	≤22	207(88.1)	28(11.9)	88(96.7)	3(3.3)	**0.25(0.08–0.85)**	**0.026**	82(92.1)	7(7.9)	0.63(0.27–1.50)	0.298
		>22	818(84.8)	147(15.2)	288(93.2)	21(6.8)	**0.41(0.25–0.65)**	**<0.001**	283(91.0)	28(9)	**0.55(0.36–0.84)**	**0.006**
	HR-HPV infection status	+	170(89.0)	21(11.0)	291(93.9)	19(6.1)	0.53(0.28–1.01)	0.054	163(91.6)	15(8.4)	0.75(0.37–1.50)	0.407
		-	352(84.2)	66(15.8)	45(95.75)	2(4.3)	0.24(0.06–1.00)	0.050	22(95.7)	1(4.3)	0.24(0.03–1.83)	0.170

Bold values show statistical data with significant difference.

*All P-values are adjusted for age, number of sexual partners, age at first intercourse, parities (including full-term pregnancy and abortion at or after 28 weeks) and age at first full-term pregnancy.

We undertook the same analysis of the distribution of Thr942Ile (942CT). There was no significant unevenness of the distribution because the *P* was <0.05 or >0.05 in the statistics for any group. Apart from the number of sexual partners in the CIN III and CSCC, there was a particularly high level of enrichment, because *P* for more than 1 group was >0.05, whereas those of the other groups were <0.05 (*P* 0.093, <0.001; *P* 0.600, 0.002, respectively). There was a significantly uneven distribution (*P* 0.298, 0.006) in the cancer group of age at first parity.

In the HR-HPV infection group, we found 844CT polymorphism in the HR-HPV-positive group had a more highly significant enrichment (*P*<0.001, 0.500, respectively) than those in the HR-HPV-negative group. Thr942Ile (942CT) polymorphism did not indicate an uneven distribution because all *P* were >0.05.

### Haplotype Analysis: Association between the Linkage Disequilibrium of the Pro844Leu and Thr942Ile Genotypes and the Risk of CSCCor CIN III

Given that the frequencies of both Pro844Leu (844CT) and Thr942Ile (942CT) genotypes change the risk of CIN III or carcinoma significantly, we next examined the linkage disequilibrium between Pro844Leu (844CT) and Thr942Ile (942CT). The frequencies of the four most common genotypes were significantly different between patients and controls. Compared to the most common genotype CC(Pro844Pro)-CC(Thr942Thr), women with the CC(Pro844Pro)-CT(Thr942Ile) genotype had a significantly decreased risk for CIN III or CSCC[OR 0.33(0.20–0.55), *P*<0.001; OR 0.60(0.40–0.91), *P* 0.017, respectively], whereas the CT(Pro844Leu)-CC(Thr942Thr) genotype increased the risk of CIN III or CSCC[OR 1.46(1.03–2.067), *P* 0.035; OR 2.04(1.48–2.80), *P*<0.001, respectively]. (**Table 4**).

**Table 4:** MLH3 genotypes and the risk of all CIN III and cervical squamous cell carcinoma.

MLH3 genotype^a^	All patients and controls							HPV-positive group						
	Control, n = 1200	CIN III, n = 400	adjusted OR^b^ (95% CI)	P	carcinoma, n = 400	adjusted OR^b^ (95% CI)	P	Control, n = 191	CIN III, n = 310	adjusted OR^b^ (95% CI)	P	carcinoma, n = 178	adjusted OR^b^ (95% CI)	P
	n(%)	n(%)			n(%)			n(%)	n(%)			n(%)		
CC-CC	917(76.4)	321(80.3)	1		288(72)	1		158(82.7)	251(81.0)	1		125(70.2)	1	
CC-CT	154(12.8)	18(4.5)	**0.33(0.20–0.55)**	**<0.001**	29(7.2)	**0.60(0.40–0.91)**	**0.017**	16(8.4)	14(4.5)	0.55(0.26–1.16)	0.116	12(6.8)	0.95(0.43–2.08)	0.894
CT-CC	108(9.0)	55(13.7)	**1.46(1.03–2.06)**	**0.035**	77(19.3)	**2.04(1.48–2.80)**	**<0.001**	12(6.3)	40(12.9)	2.10(1.07–4.12)	0.031	38(21.3)	**4.00(2.01–7.98)**	**<0.001**
CT-CT	21(1.8)	6(1.5)	0.82(0.33–2.04)	0.664	6(1.5)	0.82(0.33–2.04)	0.664	5(2.6)	5(1.6)	0.63(0.18–2.21)	0.47	3(1.7)	0.76(0.18–3.24)	0.709

Bold values show statistical data with significant difference.

a genotypes are composed of two polymorphic sites: Pro844Leu(C/T), Thr942Ile(C/T).

b All P-values are adjusted for age, number of sexual partners, age at first intercourse, parities (including full-term pregnancy and abortion at or after 28 weeks) and age at first full-term pregnancy.

Additionally, most Pro844Leu (844CT) genotypes were linkage genotype CT-CC (55/61 and 77/83) and most Thr942Ile (942CT) genotypes were linkage genotype CC-CT (18/24 and 29/35) in the CIN III and CSCC, respectively. These indicated that the majority of CT genotype distributions of MLH3 polymorphisms are caused by the linkage disequilibrium with the corresponding alleles (**Tables 2** **,** **4**). Therefore, the linkage disequilibrium between MLH3 Pro844Leu (844CT) and Thr942Ile (942CT) polymorphisms is a pivotal factor for the association with CIN III and CSCC.

In the HR-HPV-positive group, the CT-CC genotype increased the risk of CIN III and carcinoma [OR 2.10(1.07–4.12), *P* 0.031; OR 4.00(2.01–7.98), *P* 0.001, respectively]. However, the CC-CT or CT-CT haplotype was not associated with the risk of CIN III or CSCC in women with HR-HPV infection. (**Table 4**).

### Association Analysis between Haplotype and Sexual and Reproductive History in CSCC and CIN III

In the CSCC, the CC-CT haplotype might be further enriched in a particular grouping. However, it was not enriched further in the HR-HPV positive group because for both CIN III and CSCC, *P* was >0.05 when grouped according to HR-HPV infection (*P* 0.116, 0.063 and *P* 0.894, 0.195, respectively). (**Table 5**).

**Table 5:** Association between CC-CT, CT-CC genotypes and the risk for cervical squamous cell carcinoma stratified by various environmental factors.

Haplotype	High risk exposure		Controls		CIN III		Adjusted OR* (95% CI)	P	Carcinoma		Adjusted OR* (95% CI)	P
CC-CT			CC-CC(%)	CC-CT(%)	CC-CC(%)	CC-CT(%)			CC-CC(%)	CC-CT(%)		
	Number of sexual partners	≤1	737(76.5)	121(12.6)	254(80.4)	12(3.8)	**0.29(0.16–0.53)**	**<0.001**	224(72.5)	21(6.8)	**0.57(0.35–0.93)**	**0.024**
		>1	180(75.9)	33(13.9)	67(79.8)	6(7.1)	0.49(0.20–1.22)	0.125	64(70.3)	8(8.8)	0.68(0.30–1.55)	0.362
	Age at the first intercourse	≤20	275(76.6)	46(12.8)	106(81.5)	4(3.1)	**0.23(0.08–0.64)**	**0.005**	92(73.6)	5(4.0)	**0.33(0.13–0.84)**	**0.021**
		>20	642(76.3)	108(12.8)	215(79.6)	14(5.2)	**0.39(0.22–0.69)**	**0.001**	196(71.3)	24(8.7)	0.73(0.46–1.17)	0.186
	Number of parities	≤3	430(78.5)	60(10.9)	130(82.3)	3(1.9)	**0.17(0.05–0.54)**	**0.003**	92(70.2)	11(8.4)	0.86(0.43–1.70)	0.657
		>3	487(74.7)	94(14.4)	191(78.9)	15(6.2)	**0.41(0.23–0.72)**	**0.002**	196(72.9)	18(6.7)	**0.48(0.28–0.81)**	**0.006**
	Age at the first parity	≤22	186(79.1)	28(11.9)	74(81.3)	3(3.3)	**0.27(0.08–0.91)**	**0.035**	66(74.2)	6(6.7)	0.60(0.24–1.52)	0.285
		>22	731(75.8)	126(13.1)	247(79.9)	15(4.9)	**0.35(0.20–0.61)**	**<0.001**	222(71.4)	23(7.4)	**0.60(0.38–096)**	**0.034**
	HR-HPV infection status	+	158(82.7)	16(8.4)	251(81.0)	14(4.5)	0.55(0.26–1.16)	0.116	125(70.2)	12(6.7)	0.95(0.43–2.08)	0.894
		-	317(75.8)	64(15.3)	39(83.0)	2(4.3)	0.25(0.06–1.08)	0.063	19(82.6)	1(4.3)	0.26(0.04–1.98)	0.195
			CC-CC(%)	CT-CC(%)	CC-CC(%)	CT-CC(%)			CC-CC(%)	CT-CC(%)		
CT-CC	Number of sexual partners	≤1	737(76.5)	85(8.8)	254(80.4)	44(13.9)	**1.50(1.02–2.22)**	**0.041**	224(72.5)	60(19.4)	**2.32(1.62–3.34)**	**<0.001**
		>1	180(75.9)	23(9.7)	67(79.8)	11(13.1)	1.29(0.59–2.78)	0.524	64(70.3)	16(17.6)	1.96(0.97–3.94)	0.06
	Age at the first intercourse	≤20	275(76.6)	35(9.7)	106(81.5)	20(15.4)	1.48(0.82–2.68)	0.193	92(73.6)	25(20.0)	**2.14(1.21–3.76)**	**0.008**
		>20	642(76.3)	73(8.7)	215(79.6)	35(13.0)	1.43(0.93–2.20)	0.103	196(71.3)	51(18.5)	**2.29(1.55–3.39)**	**<0.001**
	Number of parities	≤3	430(78.5)	47(8.6)	130(82.3)	24(15.2)	1.69(1.00–2.89)	0.052	92(70.2)	27(20.6)	**2.69(1.59–4.53)**	**<0.001**
		>3	487(74.7)	61(9.4)	191(78.9)	31(12.8)	1.30(0.82–2.06)	0.273	196(72.9)	49(18.2)	**2.00(1.32–3.01)**	**0.001**
	Age at the first parity	≤22	186(79.1)	21(8.9)	74(81.3)	14(15.4)	1.68(0.81–3.47)	0.165	66(74.2)	16(18.0)	**2.15(1.06–4.36)**	**0.035**
		>22	731(75.8)	87(9.0)	247(79.9)	41(13.3)	1.40(0.94–2.08)	0.102	222(71.4)	60(29.3)	**2.27(1.58–3.26)**	**<0.001**
	HR-HPV infection status	+	158(82.7)	12(6.3)	251(81.0)	40(12.9)	**2.10(1.07–4.12)**	**0.031**	125(70.2)	38(21.3)	**4.00(2.01–7.98)**	**<0.001**
		-	317(75.8)	35(8.4)	39(83.0)	6(12.8)	1.39(0.55–3.52)	0.483	19(82.6)	3(13.0)	1.43(0.40–5.08)	0.580

Bold values show statistical data with significant difference.

*All P-values are adjusted for age, number of sexual partners, age at first intercourse, parities (including full-term pregnancy and abortion at or after 28 weeks) and age at first full-term pregnancy.

We analyzed the distribution of the CT-CC haplotype. According to most groupings, there was no significant enrichment besides the number of sexual partners in the CIN III and CSCC, there was a particularly high level of enrichment because the *P*-value of more than one group was >0.05, whereas those of the other groups were <0.05 (*P* 0.041, 0.524; *P*<0.001, 0.060, respectively). Meanwhile, we analyzed the HR-HPV infection grouping and we found the CT-CC haplotype, whether in CIN III or CSCC, had a more significant enrichment (*P* 0.031, 0.483; *P*<0.001, 0.580, respectively) in the HR-HPV-negative group. (**Table 5**).

### Functional Analysis

**Figure 1:**
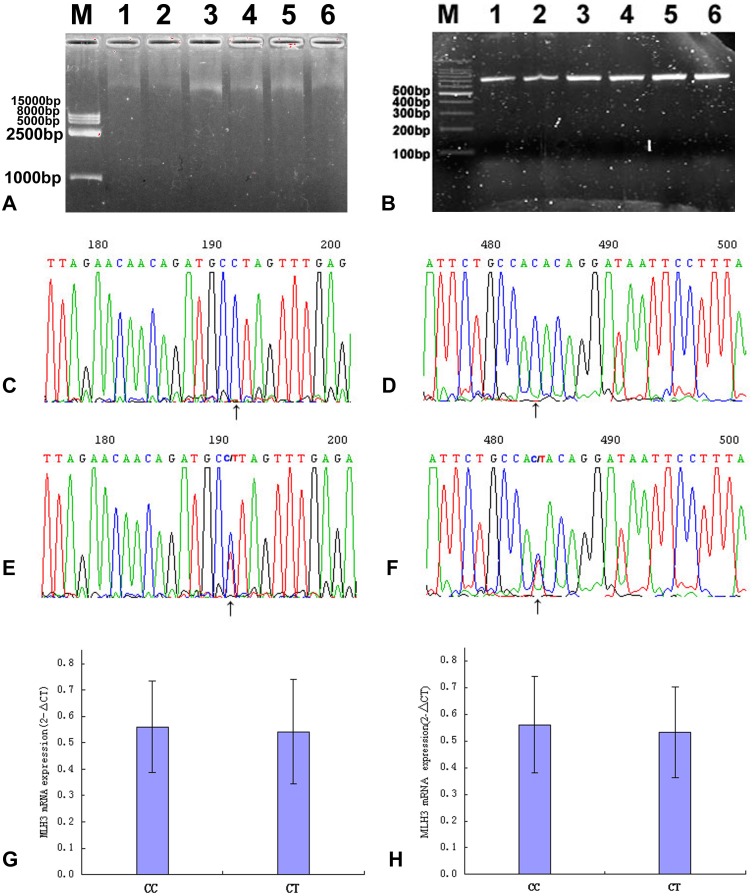
MLH3 Thr942Ile, Pro844Leu genotypes and MLH3 mRNA expression level detection. (**A**) Genomic DNA electrophoresis. M: Wide Range DNA Marker (500–15000); 1,2: Control; 3,4: CIN III; 5,6: CSCC. (**B**) Electrophoresis of PCR products. M: 100 bp DNA Ladder (Dye Plus); 1,2: Control; 3,4: CIN III; 5,6: CSCC. (**C,E**) Sequencing histogram of MLH3 Pro844Leu genotype. (**D,F**) Sequencing histogram of MLH3 Thr942Ile genotype. (**G**) Correlation of Pro844Leu genotypes with MLH3 mRNA expression levels. Mean values for Pro844Leu genotypes are: 0.561(CC), 0.543(CT). (**H**) Correlation of Thr942Ile genotypes with MLH3 mRNA expression levels. Mean values for Thr942Ile genotypes are: 0.561(CC), 0.533(CT).

The MLH3 gene mRNA expression level was assayed in tumor tissues from 282 cases of CSCC. No significant effect of MLH3 Pro844Leu or Thr942Ile genotype on MLH3 expression level was observed (*t*_Pro844Leu_ 0.670, *P*_Pro844Leu_ 0.504 and *t*_Thr942Ile_ 0.815, *P*_Thr942Ile_ 0.416) (**Figures 1G,1H**). In addition we used two transcript isoforms as input for the SIFT algorithms, the two transcript IDs were ENST00000355774 AND ENST00000238662. SIFT scores of Pro844Leu were 0.25, 0.38, respectively. SIFT scores of Thr942Ile were 0.23, 0.44, respectively. These results indicated that the impact of these two amino acid substitutions was tolerated.

## Discussion

DNA MMR inhibits tumorigenesis mainly by reducing mutations and promoting apoptosis in response to DNA damage. The highly conserved MMR proteins are able to correct DNA mismatches. MLH3 was first identified in yeast and has been mapped to the region of the mouse complex trait locus [9]. MLH3 is predicted to be involved in MMR in yeast and has an MLH1 interaction domain at its carboxyl terminus; over-expressed dominant-negative MLH3 induces MSI in mammalian cell, which indicates that it might have a role in the DNA MMR system [9].

MLH3 deficiency causes MSI, impaired DNA damage response, increased gastrointestinal tumor susceptibility and significantly shorter lifespans compared to wild-type mice [18]. MLH1–MLH3 interaction might inhibit gastrointestinal tumor initiation in mice and deficiency increased the mutation frequency of insertion–deletion loops [19]. Although pathogenic MLH3 mutation was infrequent, an inherited frameshift mutation in MLH3 was identified in HNPCC. The role of MLH3 in colorectal cancer remains controversial. Hienonen et al. [20] found 5 missense variants, 4 of which were found also in cancer-free controls; De Jong et al. [21] found two variants in Dutch patients with suspected HNPCC but did not find an association between the two variants and colorectal cancer risk. Furthermore, in a study of familial gastric cancer, no strong association was verified between 5 variants of MLH3 and gastric cancer risk [22]. MLH3 polymorphisms were shown to be associated with the risk of lung cancer and breast cancer [12], [13], and MLH3 mutation has a role in endometrial cancers [10]. These results suggest MLH exerts its role in various kinds of cancers; thus, we sought to assess whether MLH3 variants contribute to the development of CSCC and HR-HPV infection.

In this study, we found that the MLH3 Pro844Leu genotype is associated with an increased risk of CSCC, but the MLH3 Thr942Ile genotype is associated with decreased the risk of CSCC. Owing to the opposite effects of Pro844Leu and Thr942Ile variants on the risk of CSCC, we examined the linkage disequilibrium between MLH3 Pro844Leu and Thr942Ile. Compared to the CC-CC linkage haplotype, the CT-CC haplotype was at an elevated risk of CSCC. In contrast, the CC-CT haplotype decreased the risk of CSCC. Cervical carcinoma is known to develop from precursor CIN lesions through a multi-step process [23]. We observed similar association between the two SNPs in the MLH3 gene and the risk of CIN III. These indicate that the MLH3 gene maybe associated with the early molecular events of carcinogenesis.

We analyzed the enrichment of the single SNP and haplotype grouped by the number of sexual partners, age of first sexual intercourse, number of parities and age at first parity. The Pro844Leu(844CT), Thr942Ile (942CT) or haplotype were found further enrichment in some groupings, and not in other groupings according to our data. These indicated that some behavior may be affected by some genetic factors. We found obvious enrichment of Pro844Leu (844CT) and CT-CC in the HR-HPV-positive group but no further enrichment for the Thr942Ile (942CT) locus. These results indicated the Pro844Leu (844CT) locus play an important role in pathogenicity cervical cancer or precancerous lesions, and an essential role in the HR-HPV infection. The Thr942Ile(942CT) locus in the HR-HPV-positive group showed no enrichment, indicating it had an effect on the pathogenicity of cervical cancer and precancerous lesions but no effect in the HR-HPV infection process. A number of studies showed that HR-HPV positive participants had a significantly increased risk for the development of cervical carcinoma [24]. Our results suggested HR-HPV is a prime etiologic factor in cervical carcinoma but it is not sufficient for the development of cancer.

According to our results, it is plausible that the Pro844Leu genotype decreased the MLH3 function, leading to genomic instability and consequent increased susceptibility to cancer, whereas the Thr942Ile genotype might further improve MLH3 function, which implied Pro844Leu and Thr942Ile have opposite effects on MLH3 function. Although both Pro844Leu and Thr942Ile variants are related to the risk of cervical carcinoma, the CT-CC and CC-CT, but not the CT-CT, linkage haplotypes change the risk of cervical carcinoma. Thus, it is more accurate to examine the association between the linkage disequilibrium of two MLH3 SNPs and the risk of cervical carcinoma, because the function of a certain protein is determined by two or more amino acid sites [25]. These results indicate the combined analysis of the 844 and 942 sites should be included in the in vitro experiment when the domain for MLH3 protein function is investigated. The linkage disequilibrium analysis between Pro844Leu and Thr942Ile provided an important clue for the molecular mechanism of MLH3 protein function. There are functional studies of MLH3 mutation in the literature [26], [27] but there is no in vitro study of MLH3 Pro844Leu or Thr942Ile site mutation. Thus, the role of Pro844Leu and Thr942Ile in MLH3 function needs to be elucidated in vitro and in vivo.

Taylor et al. reported MLH3 Pro844Leu and Thr942Ile polymorphism will not impact MMR protein expression but predicted these two SNPs will change the structure and function of MLH3 in endometrial cancer. They concluded that MLH3 participated in the endometrial carcinoma pathogenicity by changing the function of protein MLH3 instead of altering its expression [10]. We used qRT-PCR to detect MLH3 mRNA expression in CSCC and found no difference of MLH3 expression between the CC and CT genotypes of these two SNP, and SIFT algorithm predicted there is no effect of Pro844Leu or Thr942Ile polymorphisms on MLH3 protein function, which was inconsistent with Taylor’s analysis [10]. We think this effect might be too small to be predicted by SIFT and needs the participation of other related loci. Because SIFT can calculate and predict only a single locus, the MLH3 protein function will be impacted if the two loci are changed simultaneously. In order to reveal the functional change of MLH3, we can further detect SNPs in MLH3 whole gene, or we can study the functional change after MLH3 gene specific point mutation in vitro. This is the idea that we can be of further study.
